# MicroRNA-145 regulates oncolytic herpes simplex virus-1 for selective killing of human non-small cell lung cancer cells

**DOI:** 10.1186/1743-422X-10-241

**Published:** 2013-07-22

**Authors:** Jhy-Ming Li, Kuo-Chin Kao, Li-Fu Li, Tsung-Ming Yang, Chean-Ping Wu, Yan-Ming Horng, William WG Jia, Cheng-Ta Yang

**Affiliations:** 1 Department of Thoracic Medicine, Chang Gung Memorial Hospital, 5 Fu-Hsing Street, Kweishan, 333, Taoyuan, Taiwan; 2 Graduate Institute of Animal Science, College of Agriculture, National Chiayi University, Chiayi, Taiwan; 3 Division of Pulmonary and Critical Care Medicine, Chang Gung Memorial Hospital, Chiayi, Taiwan; 4 Department of Animal Science, National Chiayi University, Chiayi, Taiwan; 5 Departments of Surgery, University of British Columbia, Vancouver, BC, Canada; 6 Department of Respiratory Therapy, Chang Gung University, Taoyuan, Taiwan

**Keywords:** Oncolytic virus, MicroRNA, Lung cancer, Radiotherapy

## Abstract

**Background:**

Non-small cell lung cancer (NSCLC) is the leading cause of cancer-related mortality worldwide, and novel treatment modalities to improve the prognosis of patients with advanced disease are highly desirable. Oncolytic virotherapy is a promising approach for the treatment of advanced NSCLC. MicroRNAs (miRNAs) may be a factor in the regulation of tumor-specific viral replication. The purpose of this study was to investigate whether miRNA-145 regulated oncolytic herpes simplex virus-1 (HSV-1) can selectively kill NSCLC cells with reduced collateral damage to normal cells.

**Methods:**

We incorporated 4 copies of miRNA-145 target sequences into the 3′-untranslated region of an HSV-1 essential viral gene, ICP27, to create AP27i145 amplicon viruses and tested their target specificity and toxicity on normal cells and lung cancer cells *in vitro.*

**Results:**

miRNA-145 expression in normal cells was higher than that in NSCLC cells. AP27i145 replication was inversely correlated with the expression of miRNA-145 in infected cells. This oncolytic HSV-1 selectively reduced cell proliferation and prevented the colony formation of NSCLC cells. The combination of radiotherapy and AP27i145 infection was significantly more potent in killing cancer cells than each therapy alone.

**Conclusions:**

miRNA-145-regulated oncolytic HSV-1 is a promising agent for the treatment of NSCLC.

## Background

NSCLC is the leading cause of cancer-related mortality worldwide [1]. Traditional treatment modalities include surgical resection, chemotherapy, radiotherapy, and targeted therapy. Patients in the early stages of the disease are better suited for surgical treatment. In advanced disease, NSCLC cells are often resistant both to chemotherapeutic agents owing to alterations in certain signal pathways [2,3] and to radiotherapy via anti-apoptotic gene overexpression [4]. Targeted therapy with receptor-tyrosine kinase inhibitors has improved progression-free and overall survival in patients with advanced NSCLC, particularly those harboring activating mutations. However, despite initial responses and long remissions, the inevitable development of secondary resistance leads to treatment failure [5]. Recently, the use of oncolytic viruses has been identified as a novel potential strategy for cancer treatment owing to its capacity to destroy tumor cells both *in vitro* and *in vivo* with minimal collateral damage to normal cells [6]. In recent decades, a number of oncolytic virus vectors have been developed with mutations in genes associated with virulence or viral DNA synthesis to confine viral replication to cancer cells and avoid causing disease [7,8]. One approach to engineering replication selectivity is the deletion of viral genes, which causes inefficient viral replication in normal cells but expansion in tumor cells. This approach was first described with herpes simplex virus type-1 (HSV-1) with thymidine kinase-negative modification, which attenuates the neurovirulence of HSV to treat human gliomas [9]. HSV-1 is a common human virus that can infect most mammalian cells. However, gene deletion might reduce the killing capability of HSV mutants in cancer *in vivo*. Another engineering approach is to place an immediate-early (IE) gene essential for HSV-1 replication under the control of a cell-specific promoter or enhancer [10]. However, our previous study [11] revealed that HSV-1 infection might up-regulate the activities of various cellular promoters and telomerase in both tumor and nontumor cells. The viral IE gene product, infected cell protein 0 (ICP0), responds by deregulating cellular promoter activity and activating telomerase. Such nonspecific up-regulation of promoter activities can result in a loss of specific control of gene expression and might cause nonselective toxicity of the HSV-1 mutant in normal human cells and tissues.

Micro-RNAs (miRNAs) are non-coding small RNAs bound to the 3′-untranslated region (3′-UTR) of targeted messenger RNAs and affect either messenger RNA cleavage or translational inhibition of genes at the post-transcriptional level [12,13]. Recent reports have suggested that miRNAs play critical roles in the tumorigenesis and progression of various human cancers [14-16]. miRNA-145 is down-regulated in several malignancies including lung cancer [14,16,17], colon cancer [18], ovarian cancer [19], and prostate cancer [20,21], and has been identified as a tumor-suppressive miRNA. Therefore, tumor-specific targeting of oncolytic HSV-1 may be achieved at the translational level by incorporating multiple copies of miRNA-145 target sequences into the 3′-UTR of an essential IE gene such as infection cell protein 27 (ICP27) or infection cell protein 4 (ICP4). Lee *et al.*[22] have used an amplicon system to show that miR-143 and miRNA-145 inhibit the expression of the *ICP4* gene at the translational level by targeting the corresponding 3′-UTR in a dose-dependent manner and thus selectively enable HSV-1 mutant replication in prostate cancer cells. In principle, this system should also permit unimpeded translation of the *ICP27* gene in lung cancer cells and subsequent oncolysis but protect normal cells owing to degradation of the amplicon transcript by miRNA-145. In the present study, we investigated the expression of miRNA-145 in normal cells and NSCLC cells and tested miRNA145-regulated ICP27 oncolytic HSV-1 for its capacity to kill NSCLC cells. We also studied the therapeutic potential of concurrent viroradiotherapy in NSCLC cells.

## Results

### Differential expression of miRNA-145 expressed in various cell lines

**Figure 1:**
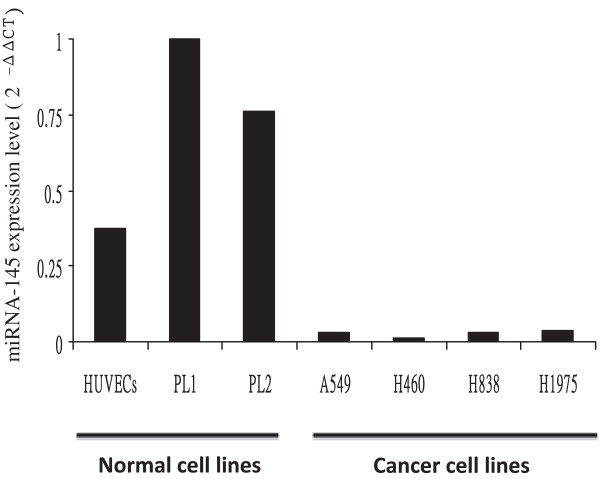
**Expression levels of microRNA (miRNA)-145 in normal cells and non-small cell lung cancer (NSCLC) cells.** Expression levels of miRNA-145 in various cell lines were determined using quantitative reverse transcription polymerase chain reaction assay. Expression levels of miRNA-145 were normalized to an internal control (miRNA-93) to obtain ΔCT values, and then all ΔCT values were compared with cells obtained from pneumonia/heart failure associated pleural effusions (PL1) to give -ΔΔCT. HUVECs, human umbilical vein endothelial cells.

miRNA-145 is reportedly down-regulated in lung cancer tissues [23,24]. To investigate the level of miRNA-145 expression in normal and lung cancer cell lines, we extracted total RNA with TRIzol® and measured the miRNA-145 expression level using quantitative reverse transcription polymerase chain reaction (RT-PCR). miRNA-145 is highly expressed in normal cells, including human umbilical vein endothelial cells (HUVECs) and cells obtained from pneumonia/heart failure associated pleural effusions (PL1 and PL2), but it is significantly down-regulated in human NSCLC cells A549, H460, H838, and H1975 (Figure 1). The miRNA-145 expression levels in HUVECs, PL2, A549, H460, H838, and H1975 were 0.376, 0.763, 0.0308, 0.01278, 0.0328, and 0.0392, respectively, relative to PL1 cells. These data indicate that miRNA-145 expression is a biomarker for differentiating normal cells and NSCLC cells.

### Expression levels of ICP27 in various cell lines after infection by AP27i145

**Figure 2:**
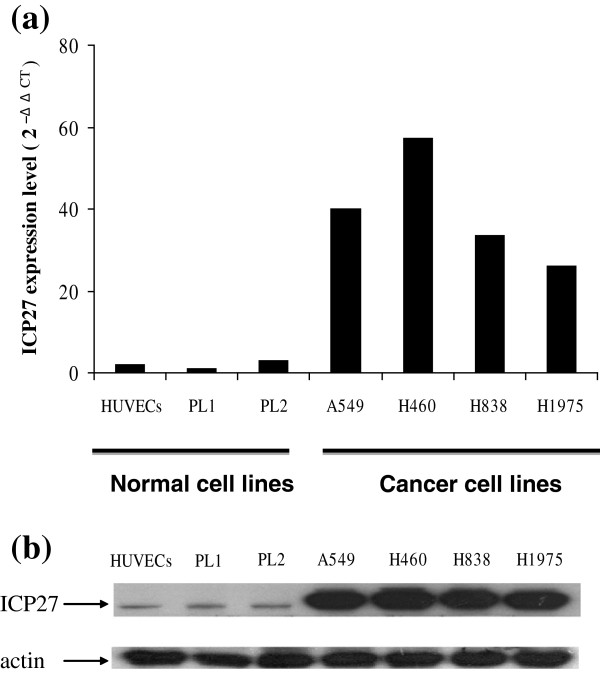
**Expression levels of ICP27 in normal cells and non-small cell lung cancer cells after infection with AP27i145.** The mRNA and protein expression levels of ICP27 in AP27i145-infected cells was determined using quantitative reverse transcription polymerase chain reaction assay and Western blot assays. **(a)** The expression of ICP27 mRNA in AP27i145-infected cells. The mRNA expression level of ICP27 was normalized to an internal control (actin) to obtain ΔCT values, and then all ΔCT values were compared with PL1 cells to give -ΔΔCT. **(b)** The expression of ICP27 protein in AP27i145-infected cells. The protein expressions of ICP27 in normal cells and non-small cell lung cancer cells after infection with AP27i145 were examined by Western blotting with antibodies specifically against ICP27 and β-actin.

Because miRNA-145 expression in NSCLC cells is lower than that in normal cells, we constructed an miRNA-145 target sequence to regulate ICP27 expression and promoted viral replication in NSCLC cells. To investigate the expression levels of ICP27 mRNA and protein, the normal and lung cancer cell lines were infected with AP27i145 at MOI of 0.1. For assaying the mRNA expression of ICP27, the total RNA of virus-infected cells was extracted with illustra RNAspin Mini Kit (GE Healthcare Life sciences; 25-0500-70) and the mRNA expression level of ICP27 was measured using quantitative reverse transcription polymerase chain reaction (RT-PCR). The result showed that ICP27 mRNA was highly expressed in human NSCLC cells A549, H460, H838, and H1975 than that in HUVECs, PL1 and PL2 (Figure 2a). The ICP27 mRNA expression levels in HUVECs, PL2, A549, H460, H838, and H1975 were 2.025, 2.84, 39.921, 57.19, 33.376, and 25.904 folds relative to that in PL1 cells, respectively. For the assessment of the ICP27 protein expression, the total proteins of virus-infected cells were extracted with protein extraction reagent and then measured by Western blotting using anti-ICP27 specific antibody. As shown in Figure 2, the protein expression levels of ICP27 were compatible with the mRNA expression levels of ICP27 in all tested cells (Figure 2b). These data indicated that the cell infected by AP27i145 could express ICP27, and the expression of viral protein ICP27 was much higher in malignant cells than in non-malignant cells.

### Comparisons of cytolytic effects between *5dl1.2* and AP27i145 HSV-1

The HSV-1 amplicon AP27i145 was generated by carrying the ICP27 gene under a cytomegalovirus (CMV) promoter with 4 copies of miRNA-145 complementary target sequences in the 3′-UTR. The amplicon plasmid also contained a viral origin of replication and packaging signal, which helped replicate and package the amplicon virus with a replication-deficient recombinant ICP27^-^ helper virus, *5dl1.2,* in host cells. The *5dl1.2* helper virus lacks the ICP27 gene and cannot replicate by itself [25]. Both cancer cells and normal cells were infected with *5dl1.2* or AP27i145 at a multiplicity of infection (MOI) of 0.001 to 0.1. The numbers of viable cells were counted 5 days after the treatments.

**Figure 3:**
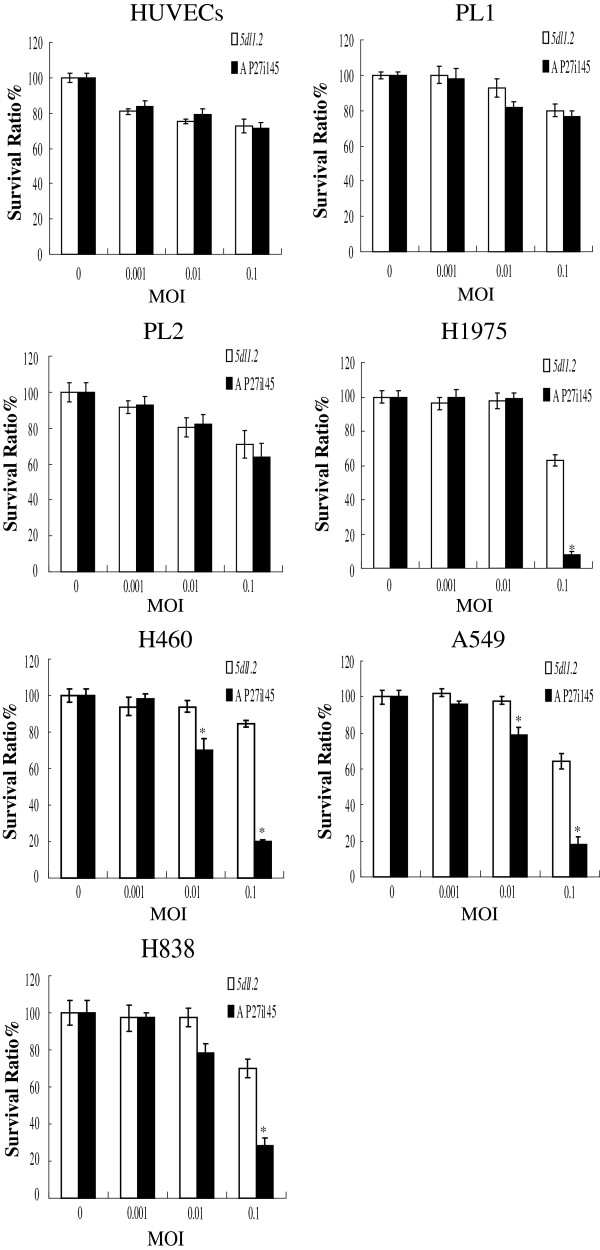
**Cytotoxicity of viral infection in normal and NSCLC cells.** Cells were treated with varying doses of AP27i145 or *5dl1.2* herpes simplex virus-1 (HSV-1). Culture medium alone was used for mock infection. Triplet cultures were performed for each treatment, and viable cells were counted on day 5. The results are expressed as a percentage of the mock-infected cells (survival ratio). Data are expressed as means ± standard error (SE). MOI, multiplicity of infection.

As shown in Figure 3, no significant difference was found in survival ratios (the ratio of viable cells in the virus-treated group to those in the mock-infected group) of in AP27i145- and *5dl1.2*-treated normal cell lines at various doses (71.2328% ± 3.6243 *vs.* 72.6027% ± 3.6243, 74.2574% ± 7.8586 *vs.* 75.2475% ± 7.9079, and 70.8333 ± 8.6736 *vs*. 63.8888 ± 1.3888 for HUVECs, PL1, and PL2, respectively; p > 0.05 in all comparisons). In A549, H460, H838, and H1975 NSCLC cells, the survival ratios of the AP27i145-infection groups were significantly lower than those of respective *5dl1.2*-infected groups at an MOI of 0.1 on post-infection day 5 (19.8260 ± 1.3386% *vs.* 84.3478 ± 1.7391%, 17.7083 ± 4.3351% *vs.* 64.5833 ± 4.1666%, 28.1578 ± 4.7441% *vs.* 70.0000 ± 4.6557%, and 7.6923 ± 2.2205% vs. 62.8205 ± 3.3919%, respectively; p < 0.05 in all comparisons). The cytotoxicity of AP27i145 was significantly stronger than that of *5dl1.2* at an MOI of 0.01 in A549 and H460 cells (69.5652 ± 2.3006% *vs.* 93.9130 ± 3.0122% and 79.1666 ± 4.1666% *vs.* 97.9166 ± 2.0833%, respectively; p < 0.05 in both comparisons; see Figure 3). This result indicated that AP27i145 caused cytotoxicity more efficiently in NSCLC cells than in normal cells.

### Correlation of miRNA-145 expression and AP27i145 replication in cells

**Figure 4:**
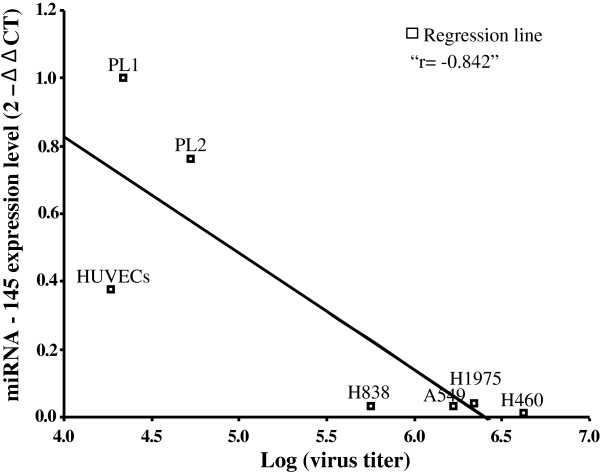
**Correlation of miRNA-145 expression and virus replication in normal and NSCLC cells.** Cells were treated with AP27i145 at an MOI of 0.1. The media were collected 5 days after treatment, and viral titer was determined using 7B cells. The correlation of AP27i145 replication and miRNA-145 expression was normalized at log 10 and calculated using SPSS software.

Because miRNA-145 expression was down-regulated (see Figure 1) and the AP27i145 HSV-1 was more cytotoxic in NSCLC cells (see Figure 3) than in normal cells, we further investigated whether the replication of AP27i145 correlated with miRNA-145 expression in cells. All tested cells were treated with AP27i145 at an MOI of 0.1. The media were collected 5 days after treatment. Using the plaque assay, we examined the virus-induced cytopathic effect on cells to determine the virus titers. The correlation of AP27i145 replication and miRNA-145 expression was normalized at log 10, and the data were analyzed using SPSS software. Figure 4 shows the strong negative correlation between the replication of AP27i145 HSV-1 and miRNA-145 expression in AP27i145-infected cells (r = -0.842). The data indicated that the AP27i145 HSV-1 replicated highly in NSCLC cells with low miRNA-145 expression.

### Inhibition of NSCLC cell growth *in vitro* by oncolytic AP27i145 HSV-1

**Figure 5:**
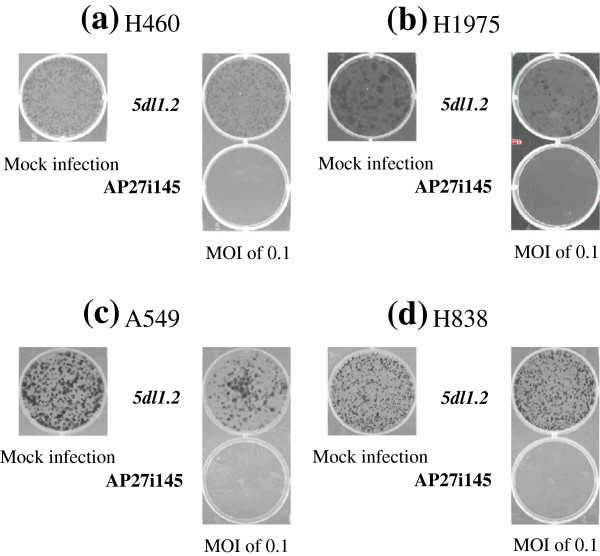
**Effects of AP27i145 infection on cell growth *in vitro*.** HSV-1 infection was performed at an MOI of 0.1. Twenty-four hours after infection, cells were seeded onto 6-well culture plates at a concentration of 10^3^ cells per well and were cultured for additional 12 days. The colonies were stained with methylene blue for photography. The experiments were performed in triplicate, and representative data are shown. **(a)** H460, **(b)** H1975, **(c)** A549, and **(d)** H838 cells.

Furthermore, we elucidated the capability of AP27i145 to inhibit NSCLC cell growth *in vitro* by assessing colony formation. We seeded A549, H460, H838, and H1975 (5 × 10^5^ cells) in 10-cm dishes and infected them with viruses at an MOI of 0.1. Twenty-four hours later, we seeded 1 × 10^3^ infected cells in 6-well plate and cultured them for 12 days. After staining the resultant colonies with methylene blue, we checked the existing colonies in the well. In contrast to the *5dl1.2*-infected groups, the AP27i145-infected groups displayed no colony growth (Figure 5). The results revealed the inhibitory capability of AP27i145 on the growth of NSCLC cells *in vitro*.

### Effects of concurrent viroradiotherapy on NSCLC cells

**Figure 6:**
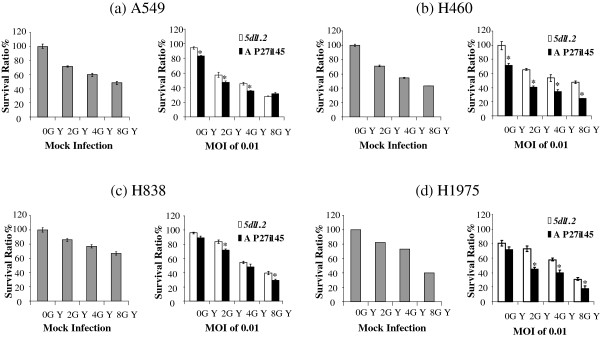
**Cytotoxicity of combined radiation and AP27i145 HSV-1 treatment. (a)** A549, **(b)**) H460, **(c)** H838, and **(d)** H1975 lung cancer cells were treated with AP27i145 or *5dl1.2* HSV-1 at an MOI of 0.01. Culture medium alone was used for mock infection. Cells were subsequently treated with radiation at 0, 2, 4, or 8 Gy 48 h after infection. On day 3 after irradiation, 3 cultures for each combination treatment were counted for viable cells. The results are expressed as a percentage of the mock-infected and mock-irradiated cells (survival ratio). Data are expressed as means ± SE.

We investigated the cytotoxicity of the combined effects of radiation and AP27i145 in NSCLC cells. A549, H460, H838, and H1975 cells were treated with *5dl1.2* or AP27i145 at an MOI of 0.01. Because the ratios of viable cell numbers in the AP27i145-infection group to those in the mock-infected group (survival ratio) were not less than 50% (78.4211 ± 4.3091%, 69.5652 ± 2.3006%, 79.1666 ± 4.1666%, and 98.71 ± 3.39%, respectively; see Figure 3), a distinctly additive efficacy was detected. Culture medium alone was used for mock infection. These cells were subsequently treated with radiation at 0, 2, 4, or 8 Gy 72 h after infection. The cells were then collected and counted on day 5. In A549 and H838 lung cancer cells, the survival ratios in the AP27i145-treated groups at 2 and 4 Gy or 2 and 8 Gy were significantly lower than those in the *5dl1.2*-treated groups (2-way analysis of variance [ANOVA], p < 0.05 in 4 comparisons); however, the survival ratios in the AP27i145-treated groups were not significantly different than those in the *5dl1.2*-treated groups at 8 or 4 Gy radiation. In H460 and H1975 lung cancer cells, the survival ratios in AP27i145-treated groups were significantly lower than those in the *5dl1.2*-treated groups from 2 to 8 Gy radiation (2-way ANOVA, p < 0.05 in 6 comparisons; Figure 6).

## Discussion

This study was proposed to determine whether we could selectively direct the oncolytic activity of a mutant herpes virus to kill NSCLC cells. miRNA-145 is reportedly down-regulated in various lung cancer tissues [26], which suggests that the 3′-UTR of miRNA-145 might regulate the expression of viral replication genes for selective targeting of tumor cells but not normal tissues. We analyzed the expression of miRNA-145 in normal and NSCLC cells using real-time quantitative RT-PCR. As expected, the expression of miRNA-145 in NSCLC cells was much lower than that in normal cells (see Figure 1). Based on this lower miRNA-145 expression pattern in cancer cells, a new class of oncolytic HSV-1 was developed and designated as AP27i145. AP27i145 is an amplicon virus carrying the ICP27 gene, an essential gene for HSV replication under the control of a CMV promoter.

Four copies of miRNA-145 complementary target sequences in the 3′-UTR of ICP27 gene may result in restricted replication of virus owing to the high miRNA-145 expression in normal cells. A significant difference was found in survival ratios between AP27i145- and *5dl1.2*-infected NSCLC cells at MOIs of 0.01 and 0.1 (see Figure 3). Accordingly, the cytotoxicity of AP27i145 at an MOI of 0.01 in H460 and A549 was significantly more efficient than that in H838 and H1975 (see Figure 3), which is consistent with the lower expression of miRNA-145 in A549 and H460 cells (see Figure 1). These data suggest that the infection of AP27i145 and the lower expression of miRNA-145 lead to considerably stronger oncolysis in NSCLC cells. Furthermore, the relatively higher expression level of miRNA-145 in normal cells significantly decreased the cytotoxicity of AP27i145, which suggests that the 4 copies of the 3′-UTR miRNA binding site sufficiently and selectively inhibit oncolytic HSV-1 replication in normal cells.

We studied the correlation between the replication of AP27i145 and miRNA-145 expression in AP27i145-infected cells. A strong negative correlation was found between the replication of AP27i145 and miRNA-145 expression in AP27i145-infected cells, confirming that the expression level of miRNA-145 may affect AP27i145 HSV-1 replication in cells. Furthermore, we demonstrated that AP27i145 selectively inhibits colony formation in NSCLC cells. The results of the present study are compatible with those of Lee *et al.*[22], who reported miRNA-145-dependent replication of the CMV-ICP4-145T virus in a prostate cancer cell line.

Oncolytic HSV can lyse infected tumor cells and spread viral progeny for further infection and killing of neighboring cancer cells. Some investigators have suggested that combining oncolytic HSV-1 treatment with ionizing radiation could increase viral replication to induce tumor cell death [27-29]. Ionizing radiation can augment the expression of the HSV-1 late gene ICP34.5 to expand viral replication. Our data indicate that the effects of combined treatment modalities including AP27i145 HSV-1 infection and radiation inhibit cell growth with significantly more potency than that of monotherapy. The cytotoxicity of combined AP27i145 HSV-1 infection and irradiation increases in a dose-dependent manner for the treatment modalities. However, the augmentation of the treatment effect via combined modalities varied by cell type.

Josson *et al.*[30] have reported that irradiation at 6 Gy decreases prostate cancer cell expression of miRNAs. Conversely, irradiation might activate p38 mitogen-activated protein kinase and up-regulate dynamin 2, thereby enhancing the activity of the CMV promoter [31]. The genetic design of AP27i145 may thus be more specific to the rise in virus production by irradiation.

In conclusion, this study demonstrates that regulating ICP27 expression with miRNA-145 can control HSV-1 to kill NSCLC cells selectively *in vitro*. The combination of this virotherapy with irradiation significantly enhanced the cytotoxicity of AP27i145 in NSCLC cells. miRNA-145-regulated oncolytic HSV-1 is a promising agent for the treatment of NSCLC.

## Methods

### Plasmid constructs

Four copies of miRNA-145 complementary sequences (miRNA-145T) were constructed as described previously [22]. The ICP27 gene (~1.8 kb) and the miRNA-145T fragments, excised by *Xho*I and *Xba*I digestion, were then cloned into the pcDNA3.0-neo vector (Invitrogen, Carlsbad, CA, USA), which contained the virus ori, viral packaging signal, and human CMV promoter to generate CMV-ICP27-145T plasmids. The ICP27 gene, including 4 copies of miRNA-145 complementary target sequences in the 3′-UTR, was controlled by the CMV promoter for expression.

### Virus recombination

The replication-deficient ICP27 helper virus (*5dl1.2*) and AP27i145 HSV-1 were packaged, propagated, and titered in 7B cells (ICP4- and ICP27-transformed African green monkey kidney cells). Monolayer 7B cells were transfected with 24 μg amplicon plasmid DNA (pCMV-ICP27-145T) using Lipofectamine 2000 (Invitrogen) according to manufacturer instructions. Twenty-four hours after transfection, cells were superinfected with *5dl1.2* virus at MOI of 1, the pCMV-ICP27-145T plasmid was thus packaged into the capsid of AP27i145 exclusively, and the virus was collected 3 days after superinfection. The AP27i145 viruses were then amplified and propagated by infecting more 7B cells. The virus titer was determined with a plaque-forming assay in 7B cells.

### Cell lines

The cell lines HUVECs, A549 (ATCC CCL-185), H460 (ATCC HTB-177), H838 (ATCC CRL-5844), and H1975 (ATCC CRL-5908) were purchased from American Type Culture Collection (ATCC; Manassas, VA, USA). The pleural effusion cell lines PL1 and PL2 were cultured from clinical specimens of 2 consecutive patients without malignancy. Cytological studies found no cancer cells in these specimens. This study was approved by the Chang Gung Medical Foundation review board, and written consent was obtained from both patients. All cells were cultured in M199, Dulbecco’s modified Eagle medium, or Roswell Park Memorial Institute medium complete medium containing 10% fetal bovine serum.

### Real-time quantification of miRNA-145 using stem-loop RT-PCR

For miRNA-145 quantification, the pulsed reverse transcription (RT) reaction described by Chen *et al.*[32,33] was performed to convert all miRNAs into corresponding complementary DNAs in a single RT reaction. Briefly, 10 μL reaction mixture containing miRNA-145-specific stem-loop RT primers (5′-CTCAACTGGTGTCGTGGAGTCGGCAATTCAGTTGAGAGGGATTC-3′, final 2 mM each), internal control-specific stem-loop RT primers (miRNA-93; 5′-CTCAACGGTGTCGTGGAGTCGGCAATTCAGTTGAGCTACCTGC-3′, final 2 mM each), 500 mM deoxyribonucleotide triphosphate, 0.5 μL Superscript III (Invitrogen), and 1 μg total RNA were used for the RT reaction. The pulsed RT reaction was performed as follows: 16°C for 30 min, followed by 50 cycles at 20°C for 30 s, 42°C for 30 s, and 50°C for 1 s. RT products were diluted 20-fold before being used for miRNA quantitative PCR. Then, 1 μL diluted RT product was used as a template for a 10-μL PCR. Briefly, 1X SYBR Master Mix (Applied Biosystems, Foster City, CA), 200 nM miRNA-145-specific forward primer (5′-CGGCGGGTCCAGTTTTCCCAGG-3′), internal control-specific forward primer (miRNA-93; 5′-CGGCGGCAAAGTGCTGTTCGTG-3′), and 200 nM universal reverse primer (5′-CTGGTGTCGTGGAGTCGGCAATTC-3′) were used for each PCR. The conditions for quantitative PCR were 95°C for 10 min, followed by 40 cycles at 95°C for 15 s and at 63°C for 32 s. All quantity PCR reactions were performed on an ABI Prism 7500 Fast Real-Time PCR system (Foster City, CA).

### Western blot

Cells (5 × 10^5^/well) were plated in 10-cm culture plates and incubated for 24 h at 37°C. After the incubation, the cells were infected with AP27i145 at MOI of 0.1 for 1 h and then all media were changed to fresh medium. After three days virus infection, the remaining cells in the dish were collected via trypsinization. The total cell lysates from virus-infected cells were extracted with lysis buffer (M-PER Mammalian Protein Extraction Reagent; 78501; Thermo Fisher Scientific Inc, Rockford, IL;) and the analysis of Western bolt were performed using mouse anti-HSV-1/2 ICP27 monoclonal antibody (sc-69806; Santa Cruz Biotechnology, Santa Cruz, CA) and goat anti-actin polyclonal antibody (sc-1616; Santa Cruz Biotechnology, Santa Cruz, CA) Horseradish peroxidase conjugated goat anti-mouse, or donkey anti-goat antibody was used as the secondary antibody (Santa Cruz Biotechnology). Chemiluminescence detection was carried out by using ECL Plus™ (GE Healthcare, Piscataway, NJ) and executed according to the manufacturer’s instructions.

### Cytotoxicity assay

Cells (1 × 10^5^/well) were plated in 6-well culture plates and incubated for 24 h at 37°C before infection. They were then mock infected or infected with either *5dl1.2* or AP27i145 at various doses (MOIs of 0.001, 0.01, and 0.1). After infection for 1 h, all media were changed to fresh media. On day 5 after infection, the remaining cells in the wells were collected via trypsinization and suspended in phosphate-buffered saline (PBS). An equal volume of 0.4% trypan blue (Sigma-Aldrich, St. Louis, MO, USA) was added to the cell suspension. Viable cells were subsequently determined via direct microscopic counting with trypan blue exclusion. All counts were carried out on 3 samples.

### Colony-forming assay

Cells (5 × 10^5^/well) were plated onto 10-cm culture dishes and incubated for 24 h at 37°C. The cells were then mock infected or infected with either *5dl1.2* or AP27i145 at an MOI of 0.1. After 1 h of infection, all media were changed to fresh media and subsequently cultured for 24 h. All cells (1 × 10^3^/well) were then seeded into 6-well culture plates and cultured for 12 days. After removal of the media, the wells were rinsed twice with PBS. Glutaraldehyde (1.25%) in PBS was added to each well, and the plates were incubated for 30 min at room temperature to allow for cell fixation. After 2 rinses with distilled water, 0.05% methylene blue solution was added to each well, and plates were incubated for 30 min at room temperature to facilitate staining of the colonies. After 2 rinses with distilled water, the plates were dried and photographed.

### Irradiation

Cells (1 × 10^5^/well) were plated onto 6-well culture plates and incubated for 24 h at 37°C before infection. Subsequently, they were either mock infected or infected with *5dl1.2* or AP27i145 at an MOI of 0.01. Forty-eight hours after infection, the cells were irradiated (0, 2, 4, or 8 Gy in a single fraction) with a 6-MeV electron beam generated by a linear accelerator (Clinac 21EX; Varian, Palo Alto, CA, USA) at a dose rate of 300 cGy min^-1^. On day 3 after irradiation, 3 cultures for each combination treatment were counted for viable cells.

### Statistical analyses

Results were expressed as means ± standard error. Statistical comparisons were made with a 2-sided *t*-test. Two-way ANOVA was used to analyze the data acquired through radiation dosing in both treatment groups. The first factor was the treatment (AP27i145 *vs. 5dl1.2* groups), and the second factor was radiation dose (0 ~ 8 Gy). A p value less than 0.05 was accepted as significant.

## Competing interests

The authors declare that they have no competing interests.

## Authors’ contributions

LJM contributed to data acquisition and analysis and drafted the manuscript; KKC contributed to data acquisition and revision of the manuscript; LLF provided technical assistance; YTM worked on aspects of the study relating to the cohort of patients with lung pleural effusion; WCP was involved in data acquisition and analysis; HYM was involved in data acquisition and analysis; JWWG provided technical assistance; YCT contributed to the study design, data analysis, and revision of the manuscript. All authors have read and approved the final manuscript.
